# Prevalence of Oral Diseases and the Influence of the Presence of Overweight/Obesity in Schoolchildren Population in Mallorca

**DOI:** 10.3390/jcm13237283

**Published:** 2024-11-29

**Authors:** Irene Coll, Daniela Vallejos, Raúl Cuesta, Jorge Domínguez, Pilar Tomás, Nora López-Safont

**Affiliations:** 1 Faculty of Dentistry, University ADEMA School, C. Passamaners 11, 07009 Palma, Spain; i.coll@eua.edu.es (I.C.); d.vallejos@eua.edu.es (D.V.); r.cuesta@eua.edu.es (R.C.); j.dominguez@eua.edu.es (J.D.); p.tomas@eua.edu.es (P.T.); 2 Health Group of University Institute for Research in Health Sciences (IUNICS), Ctra. Valldemossa Km 7.5, 07122 Palma, Spain; 3 Biology Department, University of Balearic Islands, Ctra. Valldemossa Km 7.5, 07122 Palma, Spain

**Keywords:** caries, periodontal disease, overweight, obesity

## Abstract

**Background:** The pediatric population is one of the social groups most affected by oral pathology, and overweight and/or obesity is increasingly frequently observed. This work presents a study of the prevalence of oral disease in the school population in Mallorca and its relationship with overweight/obesity. **Methods:** A cross-sectional study was carried out with a sample of 718 students aged 5–6 (*n* = 255), 12 (*n* = 230) and 15 years (*n* = 233). The WHO criteria for diagnosing and coding examined teeth and overweight/obesity prevalence values. To explore the differences in data, the mean was analyzed using the Student’s *t*-test or a one-way analysis of variance followed by the Bonferroni post hoc analysis. **Results:** Results found that students aged 15 years have a caries prevalence rate of 45.49%, higher than those aged 12 (27.39%). The presence of dental calculus in 15-year-old students is 52.8%, even higher than in 12-year-olds (30%). Students aged 6 and 12 with lower weight percentiles have fewer healthy teeth than those with higher percentiles. **Conclusions:** The schoolchildren have experienced a decrease in caries and an increase in periodontitis, with weight percentile potentially influencing the number of healthy teeth.

## 1. Introduction

Obesity and oral diseases such as dental caries and periodontal disease are growing public health issues. Although they have traditionally been addressed separately, it is recognized that nutrition is a key factor in their development. Obesity and caries are considered highly prevalent chronic diseases, multifactorial in origin, and substantially impact the lives of children and young people [1,2]. Although periodontal disease has commonly been considered a disease that affects adults, it is now affecting children and adolescents more frequently [3]. Several studies have examined the link between obesity/overweight and oral health [4,5].

Obesity nearly tripled between 1975 and 2016, with substantial increases in most countries, including low- and middle-income, which has led to it being labeled a pandemic [6]. Currently, this pandemic is centered in the United States and Europe; nearly 17% of people between the ages of 2 and 19 in the United States are obese [6].

Nationally, 40.6% of children in Spain aged between 6 and 9 are overweight or obese [7]. This statistic ranks Spain fourth in Europe for the highest prevalence of childhood obesity. In the case of the Balearic Islands, the prevalence of overweight at school age was 19.3% in 2004–2005 [8].

Oral pathologies are widespread in children and adolescents. One of the most frequent oral pathologies is dental caries [9]. This dynamic, non-communicable, multifactorial, biofilm-mediated, diet-modulated disease results in a net loss of minerals in dental hard tissues [10,11]. The development of caries is determined by biological, behavioral, psychosocial, and environmental factors [10,11], and the role of pediatricians in identifying this condition is particularly significant [12]. Another of the most common oral diseases is periodontal disease, which shares common risk factors with caries and other non-communicable diseases, such as an unhealthy diet rich in added sugars, among others [10]. Excessive intake of fermentable carbohydrates can contribute to the inflammation of periodontal tissues due to the nutritional imbalances in the microbiota [13]. Periodontal disease is a chronic degenerative disease of bacterial etiology that leads to a progressive loss of the tooth’s support structure [14].

Regarding the prevalence of oral diseases, in Spain, a third of children under 6 have caries, which represents some 850,000 students affected, amounting to nearly 4 million primary teeth with caries. One-third of adolescents have caries, representing 1.3 million young people with 2.4 million permanent teeth affected, and periodontal disease affects 25.5% of young adults today [15].

Notably, nutrition plays a relevant role in both obesity and oral pathology [16,17,18]. One of the leading causes of overweight and obesity among children and adolescents is poor eating habits, characterized by excess calories due to a high intake of fats, salt, and sugars but a low intake of vitamins, minerals, and other micronutrients. It has also been proven that sugar consumption is the main dietary factor associated with dental caries [17,19], and a deficit in the intake of micronutrients characteristic of a poor diet has been associated with a greater increase in periodontal disease [20].

Due to the correlation between nutrient excess and nutrient shortage and the onset and progression of numerous diseases, including those affecting the manifestation and development of oral pathologies, individual nutritional status plays a crucial role in multifactorial chronic diseases [16].

Another factor to bear in mind is that obesity not only leads to an increase in fat in the adipose tissue, but it is a metabolic and inflammatory disease. This has been related to the onset of inflammation in the tooth’s supporting tissues, resulting in periodontal disease [21]. Proteins called “adipokines” could be the factor linking obesity to periodontitis. These proteins are bioactive molecules with hormonal properties and a structure similar to cytokines produced by the adipose tissue [22] the greater the amount of adipose tissue, the higher the levels of leptin, interleukin IL-1 and IL-6, and tumor necrosis factor (TNF)-α. Increasing inflammatory cytokine levels in the gingival crevicular fluid hasten the progression of periodontal disease [23,24]. Hence, there is positive scientific evidence of the link between periodontal disease and overweight/obesity [23].

Obesity, characterized by an inflammatory state associated with poor nutrition (for example, excess sugar food intake), among other factors, may adversely affect oral health in schoolchildren.

For that, this study investigated the prevalence of oral disease in the school population in Mallorca and its relationship with the presence of overweight and obesity in this segment. Our study is the first to address oral health in Mallorca since the last study carried out in 2005 [22] and the first to delve into the relationship between oral pathology and overweight/obesity in this population.

## 2. Materials and Methods

### 2.1. Study Design and Target Population

This cross-sectional observational epidemiological study was designed according to World Health Organization (WHO) guidelines for conducting oral health surveys with the *Pathfinder* method [21,25]. To account for the confounding variable. An experimental design was applied prior to data collection to guarantee internal validity. This epidemiological study was designed to control the confounding variables because stratified cluster sampling was used, as per the Pathfinder method [25]. The Pathfinder method is based on the extensive methodological experience gained in oral epidemiology over the past four decades [26,27]. It is a stratified cluster sampling technique that integrates the most important population subgroups that may have varying different levels of disease. The purpose of stratification is to control the degree of confounders and create groups in which the confounder remains constant.

The school population was the target of the present study with 3 cohorts of index ages recommended by the WHO: 5–6, 12, and 15 years [25]. The sample size was calculated in the following way: for a population of 12,000 children, and a caries prevalence proportion of 0.35, a minimum sample size of approximately 340 children was required to attain a 95% confidence level with a 5% margin of error. However, we opted to increase the sample size to enhance the accuracy of our effect estimates, reduce the margin of error, and improve the statistical power of the study. Ultimately, 718 students were examined between the first year of elementary school (*n* = 255), the sixth year of elementary school (*n* = 230), and the fourth year of secondary school (*n* = 233) from selected schools on the island of Mallorca, using stratified cluster sampling.

The strata included here are population center (urban, peri-urban, and rural centers), type of school (public and charter/private), and age group (5–6, 12, and 15 years).

After segmenting the population into various strata, systematic random sampling was used to select the schools, ensuring the representativeness of each stratum by applying the proportionality criterion according to the characteristics of the study area. This included type of school: 526 students from public schools (73.3%) and 192 students from private/charter schools (26.7%); and geographic location: 404 students from urban area schools (56.3%) and 314 students from rural area schools (43.7%). Non-inclusion or exclusion criteria were applied when selecting students from the school population.

The source of information for the sampling sites (schools) was extracted from the General Directorate of Planning, Management, and Centers of the Autonomous Community of the Balearic Islands (CAIB) and the National Institute of Statistics (INE). In all the cohorts, an attempt was made to ensure that the sex variable was balanced. The study was approved by the Research Ethics Committee of the Balearic Islands (CEI: IB3737/18) per current legislation and conducted in compliance with the principles outlined in the Declaration of Helsinki and the standards of good clinical practice. Before beginning the study, information was provided to the students’ parents or guardians (they received the study information sheet and the informed consent form), and only those students whose parents or guardians signed and dated the relevant informed consent form were included.

This study was carried out in adherence to the guidelines of the Strengthening the Reporting of Observational Studies (STROBE) statement [28].

### 2.2. Data Collection and Study Variables

The data were collected between November 2018 and December 2019 using standardized lighting conditions (headlight), instruments (dental mouth mirror #5 and WHO periodontal probe), and examinee positions, for the seven dentists participating in the study, adhering to the WHO recommendations detailed in “Oral Health Surveys: Basic Methods [25].

The data collection sheet was also extracted from “Oral Health Surveys: Basic Methods” (WHO 2013) and adapted to add fields of interest for the study.

On the other hand, the dietitian-nutritionist staff participating in this study had a set of tools necessary for taking anthropometric measurements. The equipment used was a digital scale (SECA 799) with a measuring rod (220 model), an anthropometric measuring tape (SEC 203 or 201), and a skinfold caliper (brand: Harpenden Caliper). To weigh the student, the scale was placed on a stable, hard horizontal surface. The student was asked to stand right in the middle of the scale platform, with their feet slightly apart, and to remain still until the measurement was completed. Body weight was measured in kilograms, to the nearest 100 g. The student was asked to remove their shoes, and any heavy objects (phone, purse, belt, etc.) and to stay in their t-shirt and pants/skirt. The stadiometer had previously been placed on a vertical surface so that the measurement scale was perfectly perpendicular to the ground and was stable. Height was measured with the student standing, with shoulders balanced and arms relaxed along the body. Contact with the wall was maintained at five points: the back of the head, shoulders, buttocks, calves, and heels, with legs straight and feet flat, perfectly supported on the floor. The head was positioned so that the child looked forward and with the Frankfort plane parallel to the ground. If necessary, the child was helped to maintain the posture. The measurement was taken in cm, to the nearest mm. The scale, skinfold caliper, and measuring rod were calibrated and verified frequently, with a pre-check before taking measurements. The tape measure did not require calibration.

Regarding the anthropometric measurements, weight and height were to calculate the body mass index (BMI). The percentiles and hip and waist circumferences were collected because they indicate health status, while anthropometric skinfolds were used to determine body fat.

The WHO (2015) [29] tables were used as reference values for this study. Using the mean BMI of the population of the same age and sex as the benchmark, the following categories were defined:

- Underweight if the BMI < −2SD,
- Normal weight if the BMI ≥ −2SD and ≤ +1SD,
- Overweight if the BMI > + 1SD and < +2SD,
- Obese if the BMI > +2SD.

Given that our target demographic was the pediatric population, we used the percentile weight metric as [30]:

- Underweight, when the percentile weight is <5,
- Normal weight, when the percentile weight is =5 to <85,
- Overweight, when the percentile weight is =85 to <95,
- Obese, when the percentile weight is ≥95.

### 2.3. Analyzed Variables

- For the analysis of sociodemographic variables, the following variables were considered: sex, age.
- For the analysis of caries prevalence, the following variables were considered:
  dmf index (sum of decayed, missing and filled primary teeth), DMFT index (sum of decayed, missing, and filled permanent teeth).
- For the analysis of periodontal disease prevalence, the following variables were considered: community periodontal index (CPI), and number of sextants affected.
- For the analysis of obesity prevalence, the following variables were considered: weight, percentiles, fat, waist circumference, hip circumference, waist-hip ratio (WHR), waist-to-height ratio, and body mass index (BMI).
- For the analysis of the relationship/association between obesity and oral pathology, the following variables were considered: number of healthy teeth.

### 2.4. Consistency and Reproducibility of the Data

To determine the prevalence of oral disease among the children and adolescents studied using the oral and dental examinations carried out and to ensure consistency and reproducibility in the data analysis, the recommendations outlined in the manual “Oral Health Surveys: Basic Methods” [25] were followed. Per these recommendations, a pilot intervention, consisting of a four-day Monitoring Conference, was conducted (Table 1).

This pilot intervention was necessary for the application of the examinations needed to ensure inter-rater agreement (between one examiner and another), resulting in 0.757. Based on the inter-rater agreement data, the simple agreement percentage [(coincident diagnoses/total examiner) × 100] and the Kappa index are calculated using the Landis and Koch scale [31], being 98.7% and 0.757, respectively.

To determine the reliability of the measurements, a Gold Standard (an experienced examiner) was evaluated as a reference, measuring the degree of agreement of each examiner compared to the Gold Standard using the Kappa index calculation.

As noted, the mean Kappa values (0.757) are over 0.61, indicating a high level of agreement (Table 2). The levels of simple agreement are over 95% (98.7%) and between 85 and 95%. These percentages are considered adequate to begin the study (Table 2).

### 2.5. Ethical Treatment of the Data and Statistical Analysis

The data are computerized and input into an automated record of personal data collected for the study. The collected data were anonymized to guarantee confidentiality.

The data were analyzed with the statistics application SPSS 27.0.1.0^®^. In terms of the type of variable and the groups to be analyzed, the differences in data were determined using the analysis of the mean by the student *t*-test or a one-way analysis of variance (ANOVA) followed by the Bonferroni post hoc analysis. In each case, the 95% confidence interval estimate was used (*p* < 0.05) to have an accurate measurement (of the random error present in the data). Multiple regression (adjusted for sex, age, area of residence, and type of school) was applied to estimate the odds ratios (ORs) and 95% confidence intervals (CIs) of oral pathology (caries and periodontal disease) by overweight/obesity adjusted for potential confounders, this analysis was used to determine effect size. All the tests were two-sided, and the significance level was 0.05.

## 3. Results

Descriptive statistics of the participants in this study. In this study, we analyzed 381 boys and 337 girls in 1st and 6th grades elementary and 4th year secondary; therefore, they were classified according to age (5–6 years in 1st grade elementary, 12 years in 6th grade elementary, and 15 years in 4th year secondary school). Of the sample, 53.0% were boys and 46.9% were girls. Regarding the area of residence, 22.5% of the sample was from urban and peri-urban areas, 50.4% from rural areas, and 27.02% of the sample was unknown. Concerning the country of birth, 86.90% of the sample was Spanish, 9.9% was from other nationalities, and 3.20% was unknown. Regarding the type of school, 73.3% of the sample was public schools, and 26.7% were private/charter schools. (Table 3). We have analyzed the prevalence of diseases in the total sample.

### 3.1. Analysis of Caries Disease

Prevalence of caries by age: The prevalence of caries (dmf or DMFT > 0) for each cohort is detailed in Table 4, showing that at the age of 5–6 years, the percentage of students affected with primary teeth dmf > 0 is 40.00% and permanent teeth with DMFT > 0 is 7.84%. At 12 and 15 years, the percentage of teeth with a history of caries in the permanent dentition is 27.39% and 45.49%, respectively (Table 4).

**Figure 1:**
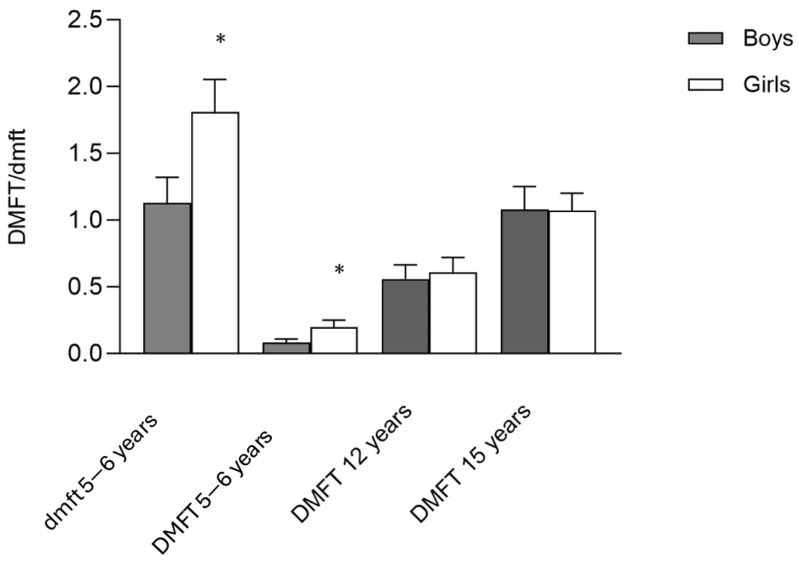
DMFT or dmf index in the school population. The results represent means ± SD. Bars with * are significantly different (one-way ANOVA, *p* < 0.05 and Bonferroni post hoc analysis). No * = no significant difference. (dmf, decayed, missing, and filled primary teeth; DMFT, decayed, missing, and filled permanent teeth).

Caries Index by age: Figure 1 presents the DMFT indices in the different cohorts and dmf in the 5–6-year-old cohort. Note that the dmf is higher in girls than in boys (boys, 1.13 ± 2.30 vs. girls, 1.81 ± 2.50, *p* = 0.014), and the DMFT index in the 5–6- and 12-year-old groups is higher in girls than in boys (5–6 years: 0.20 ± 0.63 vs. 0.08 ± 0.043, *p* = 0.044); 12 years: 0.60 ± 1.16 vs. 0.55 ± 1.17, *p* = 0.415) unlike the 15-year-old group, where the DMFT index is slightly higher in boys than in girls (1.08 ± 1.83 vs. 1.07 ± 1.47, p=0.489).

### 3.2. Distribution of Caries

Table 5 shows the sample distribution in each of the three age groups in terms of dmf or DMFT. In the primary dentition, at the age of 5–6, the percentage of subjects without caries is 60.0%. In the permanent dentition, this value is 92.2% (5–6 years), 72.6% (12 years), and 55.2% (15 years).

### 3.3. Cumulative Distribution of the DMFT Index

In a disease like caries, which has an asymmetric distribution, it is equally as important to look at the distribution as the mean. Most lesions are concentrated in a low percentage of individuals. Thus, at 12 years of age, 72.6% are caries-free, while 14.8% of students have 78.7% of the total number of teeth affected by caries. At 15 years of age, 54.5% are caries-free. Similarly, 27.7% of the sample have 82.9% of teeth affected by caries (Table 6).

### 3.4. Analysis of Periodontal Diseases

#### Prevalence of Periodontal Disease by Age

Table 7 shows the percentages of subjects in each maximum CPI (Community Periodontal Index) code. This index was only collected from 12 years of age onward. In the 12- and 15-year-old cohorts (only the presence of dental calculus and bleeding on probing are assessed), the percentage of individuals with calculus or tartar is 30.0% and 52.8%, respectively. By contrast, a healthy periodontium is found in 30.0% and 22.7%.

**Figure 2:**
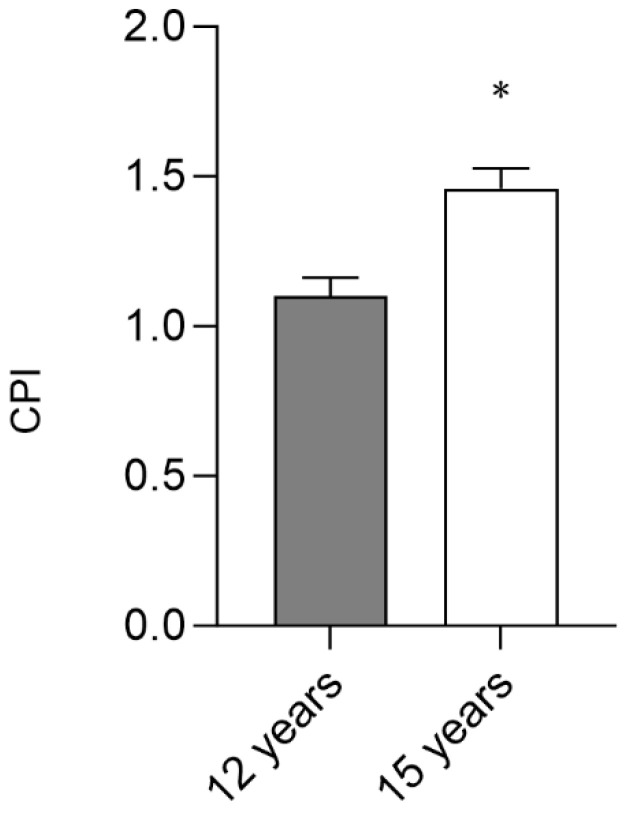
Mean of the Community Periodontal Index. The results represent means ± SE. Bars with * are significantly different (Student’s *t*-test analysis, *p* < 0.05). (CPI; community periodontal index).

As noted in Figure 2, there are significant differences between the mean of the community periodontal index at 12 years (1.10 ± 0.06) and 15 years (1.46 ± 0.06;p<0.001).

Number of affected sextants by age: In relation to the severity of the process (Table 8), a significant difference is observed in the 12-year age group, with the mean in the group of girls being higher than in the group of boys (1.86 ± 2.00 vs. 1.21 ± 1.56; *p* = 0.004).

**Figure 3:**
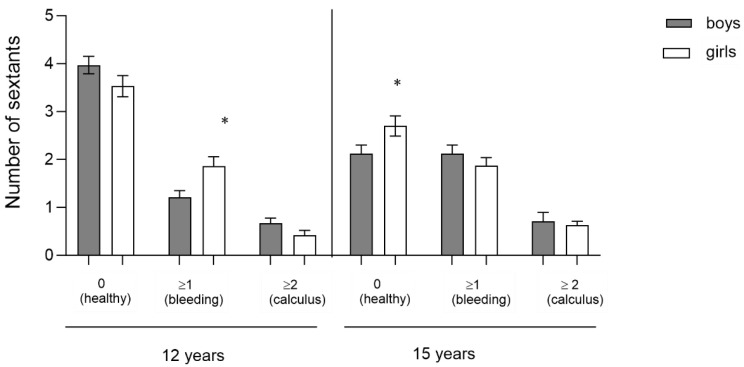
Mean of the Community Periodontal Index (12–15-year-old). Mean number of affected sextants. The results represent the means ± SE. Bars with * are significantly different (one-way ANOVA, *p* < 0.05 and Bonferroni post hoc analysis). No * = no significant difference.

As noted in Figure 3, there are significant differences in the 12-year age group, where girls have a higher mean number of bleeding sextants ≥1  than the boys (1.86 ± 0.20 vs.  1.21±0.14; *p* = 0.004). In the 15-year-old group, girls have a higher mean number of healthy sextants than boys the same age (3.26 ± 0.20 vs. 2.70 ± 0.21; *p* = 0.029).

Prevalence of overweight/obesity by age: The prevalence of overweight/obesity by age is detailed in Table 9, showing that the presence of overweight and obesity is higher in boys in all age cohorts.

At the age of 5–6 years, 39.04% are overweight/obese, 60.16% have normal weight, and 0.80% are underweight. In the 5–6 age group, male students with overweight/obesity represent 44.37%) and female students 32.11%. 54.23% of male students and 67.89% of female students have a normal weight. Male underweight students represent 1.41% and 0.0% in the case of female students.

At the age of 12 years, 43.17% are overweight/obese, 53.74% are normal weight, and 3.08% are underweight. At the age of 12 years, male students who are overweight/obese represent 44.80% and female students 41.18%. In the case of normal weight, male students represent 53.6% and female students 53.92%, and in the case of underweight, male students represent 1.60% and female students 4.90%.

At the age of 15 years, 29.74% are overweight/obese, 68.53% are normal weight, and 1.72% are underweight. At the age of 15 years, male students who are overweight/obese represent 31.25% and girls 28.33%. In the case of normal weight, male students represent 66.07% and female students 70.83%, and in the case of underweight, male students represent 2.68% and female students 0.83%.

**Figure 4:**
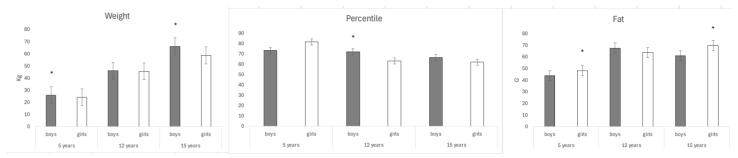
Means of weight, percentile and fat (5/6 -12–15-year-old The results represent the means ± SE. Bars with * are significantly different (one-way ANOVA, *p* < 0.05 and Bonferroni post hoc analysis). No * = no significant difference. (Kg, kilogram; G, gram).

Figure 4 shows the means of weight, percentile, and fat by age and sex. Note that the percentage of subcutaneous fat is higher in the girls than the boys in the 5–6-year-old cohort (48.08 ±16.98 vs. 43.89±17.85 p=0.030) and in the 15-year-old cohort (69.91 ± 24.45 vs. 60.90±25.10 p=0.003). There are significant differences with respect to the mean percentile in the 12-year cohort (boys 71.93 ± 26.81 vs. 63.16 ± 28.87; *p* = 0.009). Significant differences are also found in the mean weight in the 5–6-year-old cohort (boys 25.84 ± 9.25 vs. girls 24.23 ± 4.63; *p* = 0.049) and in the 15-year-old cohort (boys 66.07 ± 11.99 vs. girls 58.68 ± 11.03; *p* ≤ 0.001).

**Figure 5:**
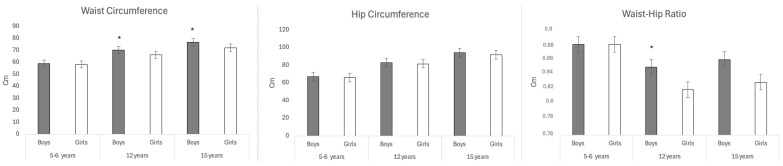
Mean of waist circumference, hip circumference, and waist-hip ratio (5/6 -12–15-year-old). The results represent the means ± SE. Bars with * are significantly different (one-way ANOVA, *p* < 0.05 and Bonferroni post hoc analysis). No * = no significant difference. (Cm, centimeter).

There are significant differences with respect to the mean waist circumference in the 12-year-old cohort (boys 70.33 ± 10.46 vs. girls 66.17 ± 11.86; *p* = 0.003) and in the 15-year-old cohort (boys 76.82 ± 10.22 vs. girls 72.26 ± 13.34; *p* = 0.002) as can be seen in Figure 5 with boys at both ages having a larger circumference than girls with a difference of 5.9% at both 12 and 15 years of age.

The measurement of waist circumference (WC) can be useful information for assessing cases of excess weight in which abdominal adiposity is evident and may pose an added cardiovascular or metabolic risk. It is observed that the mean waist-hip ratio (WHR) is 7% higher in boys at the age of 12 than in girls (boys 0.85 ± 0.13 vs. 0.82 ± 0.01; *p* = 0.013). Recent studies confirm that the WHR is the most accurate anthropometric indicator to be considered in assessing total body fat and intra-abdominal fat mass. We did not observe significant differences in the mean hip (Figure 5).

Table 10 provides the mean data and percentiles of the parameters measured and assessed for the total population and by age group.

### 3.5. Interrelationship of Oral Health and Physical Condition in Students

No association was noted between overweight/obesity and oral pathology (caries: OR = 1.179, 95% CI (0.715–1.943), *p* = 0.518/periodontal disease: OR = 1.085, 95% CI 95% (0.698–1.686), *p* = 0.714) (Table 11 indicates the multivariate regression models for the association between overweight/obesity and oral pathology, adjusted for potential confounders).

**Figure 6:**
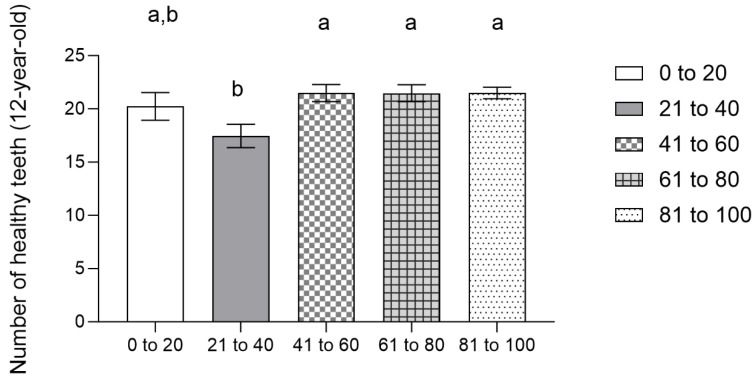
Mean number of healthy teeth according to weight percentile (12-year-old). The results represent means ± SD. Bars that do not share a letter (a, b) are significantly different (one-way ANOVA, *p* < 0.05, and Bonferroni post hoc analysis).

However, when we analyze healthy teeth according to weight percentile, we observe that 12-year-old students with weight percentiles between 20 and 40 have significantly fewer healthy teeth than students in a percentile above 40 (0 to 20: 20.2308 ± 4.7461; 21 to 40: 17.4615 ± 6.8200; 41 to 60: 21.4848 ±4.66450; 61 to 80: 21.4667 ± 5.48696; 81 to 100: 21.4948 ± 5.48696; *p* = 0.003) (Figure 6).

**Figure 7:**
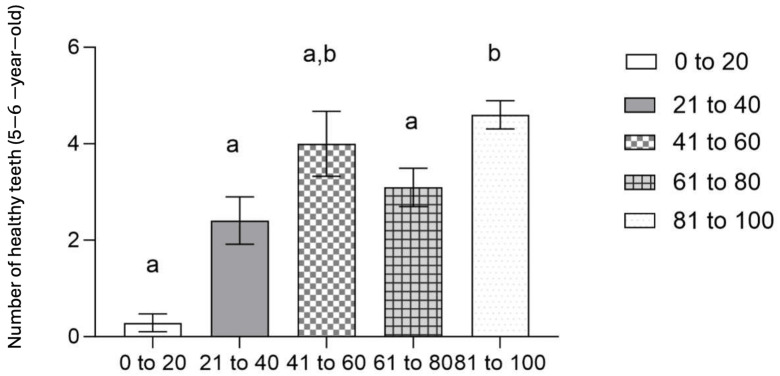
Mean number of healthy teeth according to weight percentile (5–6-year-old). The results represent means ± SD. Bars that do not share a letter (a, b) are different (one-way ANOVA, *p* < 0.05 and Bonferroni post hoc analysis).

Additionally, in Figure 7, we observe that in 6-year-old students the number of healthy teeth is conditioned by the percentile range of the students, with a greater number of healthy permanent teeth observed with higher percentile ranges (0 to 20: 0.2857 ±0.18443; 21 to 40: 2.40742 ± 0.48995; 41 to 60: 4.000 ±0.67320; 61 to 80: 3.0952 ± 0.39565; 81 to 100: 4.5984 ±0.29277; *p* = 0.000).

Therefore, in general, in schoolchildren aged 6 and 12, a pattern was noted indicating that the lower the weight percentiles, the lower the number of healthy teeth compared to the students with the highest weight percentiles. However, this pattern is not observed in the 15-year-old cohort.

## 4. Discussion

The increasing prevalence of oral diseases, such as caries and periodontal diseases, and their association with childhood obesity have been recognized globally as interrelated public health problems. However, in Mallorca, no previous study had been carried out that specifically addressed this relationship, which further justifies the need for this analysis of the island’s school population.

This study is based on a cross-sectional observational study, the design of which is ideal for studying the prevalence of chronic diseases like caries and periodontitis [32], and the influence of the presence of overweight/ obesity, specifically in this case, in the child and adolescent population in Mallorca.

Studying variables such as dental caries, periodontal disease and obesity in the child and youth population is of utmost importance due to their direct impact on short- and long-term health [33].

The child and adolescent population’s unhealthy lifestyles [18] due to the dynamism of modern life are well known, leading, in many cases, to an increased presence of overweight and obesity in this population. Therefore, we sought to establish a possible link between a systemic pathology like obesity and the onset of oral pathology among children and adolescents.

The target population of this study is school-aged, as this is one of the social groups most affected by oral pathology in general [34]. It has been observed among the population in developed countries that overweight and/or obesity are increasingly observed at younger ages [35]. Therefore, obesity and caries (as the main oral disease, followed by periodontitis) are two of the main diseases with the highest prevalence among children and adolescents, becoming a significant public health issue worldwide in recent decades [36,37].

In the last oral health survey in Spain conducted in 2020 [38], the percentage of dmf > 0 (sum of decayed and filled primary teeth) in students aged 5–6 years was 35.50%, slightly higher than in the 2015 survey [38,39]), which was 31.50%. In our study, the percentage of dmf > 0 was 40.00%, higher than the 2020 state survey but lower (45.5%) than the latest oral health survey in the Balearic Islands [40]. Therefore, we could say that the prevalence of caries among elementary students in Mallorca is lower.

Regarding the % DMFT > 0, in 12-year-old students, there is a clear trend toward a decrease in caries, as the percentage has decreased in all national and provincial surveys. Specifically, at the national level, the %DMFT > 0 at 12 years has decreased from 33.30% to 28.60% in 5 years [39,40]. In the Balearic Islands, the trend is the same, dropping from 34.9% to 27.9% (data reflected in our study) in 10 years [41].

This is not the case in the 15-year-old cohort, where in our study, the percentage of DMFT > 0 is much higher than in the latest state oral health survey [38,39,40]; specifically, these students in Mallorca have a caries prevalence of 45.49%, which is higher than the 2020 national mean of 35.50%. However, there is also a decrease from the last record in the Balearic Islands in 2005. Back then, it was 60.2%, while in our study, it was 45.49%. Therefore, there is a downward trend of caries prevalence at age 15 among students in Mallorca.

These data are also reflected in the calculation of the caries index. The caries index (dmf and DMFT) in all the cohorts has seen a reduction in percentage from the last study in the Balearic Islands [40] to the recent study of students in Mallorca (2018). However, the dmf index in 2018 was 1.4275, much higher than the 2020 survey, which was 1.28, and the 2015 survey, which was 1.11. The caries index at 12 years (0.67) and 15 years (1.07) presents a higher percentage in the Mallorca study (2018/2019) than in the 2020 survey (12 years: 0.58/ 15 years: 0.94), but lower than in the 2015 survey (12 years: 0.71/15 years: 1.34).

A decrease in the prevalence of caries was observed, both in primary and permanent dentition, across all age groups, compared to the last local study [40]. However, despite a decline in the 2018–2005 period, the prevalence of primary dentition is still higher than that in the first local study [41].

In the 2005 oral health study, the prevalence of tartar was 31% among 14-year-olds and 19% among 12-year-olds [40]. Our study revealed that the percentage of tartar or dental calculus was higher than in the 2005 survey, but also higher in the 15-year-olds at 52.8% than the 12-year-olds at 30.0%, with a greater prevalence in the boys. Most notably, only 22.7% of 15-year-olds in our study had a completely healthy periodontal status, a figure lower than the 2020 oral health survey, with 36.7%, and much lower than the 2015 survey with 46.0%.

On the other hand, regarding the mean number of sextants with bleeding, there are significant differences at 12 years in terms of sex, both in the oral health survey (2020) and in the survey of students in Mallorca, with girls being higher in both cases than boys. The 2005 oral health survey did not mention gender differences in the mean number of sextants with bleeding, only the mean number of sextants with tartar, which was higher in male students [40].

The prevalence of periodontal disease has increased as evidenced by a reduction in schoolchildren with healthy sextants (without periodontal disease) and an increase in those exhibiting sextants with bleeding and sextants with calculus (with periodontal disease) compared to, the last local study [40].

On the other hand, in the Balearic Islands, the first study on the Prevalence of Childhood and Adolescent Obesity (EPOIB) conducted during the 2004–2005 school year published data on the prevalence of overweight being 19.3% (overweight 10% and obesity 9.3%) [8,42].

In the second prevalence study on childhood and adolescent obesity in the Balearic Islands 2016–2017 [42], obesity in the Balearic Islands was 10.4%. 25% of the students were overweight (overweight plus obesity), with a higher frequency among boys. In our sample, 63.10% of the study population had a normal weight, 23.11% were overweight, and 13.79% were obese. In our sample of schoolchildren aged 5–6 and 15 years, weight was higher in boys than in girls. Among schoolchildren aged 12 years, there were no differences between the sexes. On the contrary, the percentage of subcutaneous fat was higher in girls than in boys in schoolchildren aged 5–6 and 12 years.

Significant differences were also found regarding the mean waist circumference in the 12-year-old cohort and in the 15-year-old cohort, with boys at both ages having a greater circumference than girls by a difference of 5.9% at both 12 and 15 years.

It was also observed that the mean waist-hip ratio (WHR) was 7% higher in boys at the age of 12 years than in girls.

The prevalence of overweight and obesity has increased compared to the last local study [42], and boys present higher figures in the 5–6 and 15-year-old cohorts.

Currently, some authors propose a link between overweight/obesity and caries in children [43,44], while other authors indicate no such association exists [45]. In our study, the results suggest that there is no association between overweight/obesity and oral pathology (caries and periodontal disease). However, we observed that the number of healthy teeth is conditioned by the percentile range of the students in the 5–6- and 12-year-old cohorts. These results require deeper investigation but could be linked to poor nutrition, possibly because they are undernourished (malnutrition) and consequently have a lower intake of the vitamins needed for good oral health [46].

However, at the age of 15, a trend is observed suggesting that obesity may be a risk factor for the prevalence of dental caries. This could indicate that there may be differences in health patterns and associated risk factors at different stages of child and adolescent development. This finding could be interpreted as a shift in the factors influencing oral health as children approach adolescence and, eventually, adulthood.

The latest studies report evidence of a direct link between gum disease (periodontal disease) and overweight/obesity in adults [47]. It is known that adipose tissue not only acts as a fat reservoir but is also an endocrine organ capable of secreting pro-inflammatory molecules such as leptin, interleukin-6, TNF-alpha, etc. These adipokines play a key role in inflammation. And this can worsen oral health in terms of increased gum inflammation [16]. Specifically, in our study, we noted that the student’s gender influenced the prevalence of the number of sextants: 12-year-old female students have 0.87% more sextants with bleeding than male students of the same age, but we found no relationship with obesity in the pediatric population studied. It is known that alterations in sexual hormone levels are more frequently linked to girls than boys because estrogen and progesterone are predominant in the female sex [48].

Poor nutrition can affect children’s oral health, and above all, the impact of the lack of dental hygiene is very important in oral pathology [49]. Intervention at early ages through motivational interviewing to promote a change of habits in the family and psychological support is vital in the approach to the problem, without neglecting nutritional updating, dispelling myths, and encouraging increased physical activity [50].

Historically, several studies have examined the link between obesity/overweight and the presence of oral health with varying results. The great challenge is to understand and consider possible confounding factors (diet and socioeconomic level) and modifying effects (age, oral hygiene, fluoride use) in a systematized way [4,5].

Due to the type of design, the main weakness of this study is that there is no follow-up, and this leads to limitations when it comes to establishing a causal relationship. Furthermore, in our study, the associations observed do not imply direct causality, although the correlations found may be interesting and generate future hypotheses. Concretely, the students in this study were classified as obese or overweight and normal weight (as a control group) to determine the link between oral pathology and obesity. Also, socioeconomic status and dietary habits were not analyzed in this study, and these could contribute relevant information. Conversely, a primary strength is the straightforward applicability of the findings attributable to the extensive representative sample of the schoolchildren population. Another strength of this study is the exhaustive analysis of all the variables related to obesity in the school population and oral pathology.

## 5. Conclusions

A decrease in the prevalence of caries and an increase in periodontal disease were observed in the school population in Mallorca compared to previously recorded data, and obesity prevalence increased slightly. Additionally, the presence of oral pathology (caries and periodontal disease) seems to relate to age; the older a person is, the greater the presence of caries and periodontal disease. Gender may influence the prevalence of periodontal disease, whereas weight percentile range could affect the proportion of healthy teeth in the pediatric population.

Further research is warranted, including an analysis of students’ dietary habits, to obtain more conclusive evidence on the incidence of these factors and the development of dental pathology and overweight/obesity.

## Figures and Tables

**Table 1:** Monitoring Conference.

Day 1	Presentation of the survey. Contact with research personnel. Presentation of the instrumentation: lighting and examination equipment Exploration of the clinical record: how to fill it in Clinical criteria according to WHO standards for each of the study parameters Viewing and discussion of examples
Day 2 and Day 3	Calibration of the practice on students: performing cross scans for the analysis of inter-rater agreement needed to calculate the Kappa index
Day 4	Discussion and assessment of agreement. Logistical instructions for sampling, parental (student) permission, data submission

**Table 2:** Dental status: Analysis of inter-rater diagnostic agreement, taking the tooth as the unit of analysis. (SE: Standard error).

Examiner	Kappa ± SE
Examiner 001	0.51 ± 0.14
Examiner 002	0.68 ± 0.10
Examiner 003	0.63 ± 0.10
Examiner 004	0.85 ± 0.06
Examiner 005	0.88 ± 0.20
Examiner 006	0.79 ± 0.08
Examiner 007	0.94 ± 0.08

**Table 3:** Distribution of the students by grade, sex, area of residence, country of birth, and type of school. (N, sample size; USA, United States of America).

		1st Grade Elementary (5–6 Years)		6th Grade Elementary (12 Years)		4th Year Secondary (15 Years)		Total	
		N	%	N	%	N	%	N	%
Sex	Male	144	56.47	125	54.35	112	48.06	381	53.06
	Female	111	43.53	105	46.65	121	51.93	337	46.94
Area of Residence (from student)	Urban +Peri-urban	47	18.43	36	15.65	79	33.90	162	22.56
	Rural	158	61.96	120	52.17	84	36.05	362	50.42
	Known	205	80.39	156	67.83	163	69.95	524	72.98
	Unknown	50	19.61	74	31.17	69	29.61	193	27.02
Country of Birth (from student)	Spain	220	86.27	203	88.26	201	86.26	624	86.90
	Other countries of the European Union	7	2.75	12	5.22	8	3.45	27	3.77
	Canada + USA	1	0.39	0	0	1	0.43	2	0.28
	Rest of America	8	3.14	8	3.48	9	3.88	25	3.49
	Asia	3	1.18	0	0	5	2.16	8	1.12
	Africa	3	1.18	5	2.17	1	0.43	9	1.26
	Oceania	0	0.00	0	0	0	0.00	0	0.00
	Known	242	94.90	228	0	225	96.56	695	96.79
	Unknown	13	5.10	2	0.87	8	3.43	23	3.20
Type of school	Public	177	69.4	159	69.1	191	81.6	527	73.3
	Private/ Charter	78	30.6	71	30.9	43	18.4	192	26.7

**Table 4:** Distribution of students with caries experience in primary and permanent dentition: dmf or DMFT > 0. (N, sample size; SD: standard deviation; CI, confidence interval; dmf, decayed, missing, and filled primary teeth; DMFT, decayed, missing, and filled permanent teeth).

		N	Mean	SD	%	(95%CI)
5–6 years	Primary dentition	102	1.47	2.44	40.00	(33.9–46.01)
	Permanent dentition	20	0.13	0.53	7.84	(4.54–11.1)
12 years	Permanent dentition	63	0.58	1.16	27.39	(21.6–33.1)
15 years	Permanent dentition	106	1.08	1.65	45.49	(39.0–51.8)

**Table 5:** Caries index. Details of the dmf or DMFT distribution. (CI, confidence interval; dmf, decayed, missing, and filled primary teeth; DMFT, decayed, missing, and filled permanent teeth).

5–6 years (primary)	*n* (255)	%	(95% CI)
dmf = 0	153	60.0	(54.0–66.0)
dmf = 1	27	10.6	(6.8–14.4)
dmf = 2	20	7.8	(4.5–11.1)
dmf = 3	14	5.5	(2.7–8.3)
dmf = 4	11	4.3	(1.8–6.8)
dmf = 5–9	27	10.6	(6.8–14.4)
dmf ≥ 10	3	1.2	(0.0–2.5)
5–6 years (permanent)	*n* (253)	%	(95% CI)
DMFT = 0	235	92.2	(88.9–95.5)
DMFT = 1	11	4.3	(1.8–6.8)
DMFT = 2	6	2.4	(0.5–4.2)
DMFT = 3	1	0.4	(0.0–1.2)
DMFT = 4	2	0.8	(0.0–1.9)
DMFT = 5–9	0	0.0	(0.0–0.0)
DMFT ≥ 10	0	0.0	(0.0–0.0)
12 years	*n* (230)	%	(95% CI)
DMFT = 0	167	72.6	(66.8–78.4)
DMFT = 1	29	12.2	(8.3–16.9)
DMFT = 2	14	6.1	(2.7–8.6)
DMFT = 3	8	3.5	(1.1–5.8)
DMFT = 4	9	3.9	(1.4–6.4)
DMFT = 5–9	2	0.9	(0.0–2.8)
DMFT ≥ 10	1	00.4	(0.0–1.3)
15 years	*n* (245)	%	(95% CI)
DMFT = 0	127	55.2	(48.8–61.6)
DMFT = 1	43	18.7	(13.7–23.7)
DMFT = 2	27	11.7	(7.6–15.9)
DMFT = 3	12	5.2	(2.3–8.1)
DMFT = 4	12	5.2	(2.3–8.1)
DMFT = 5–9	11	4.8	(2.0–7.5)
DMFT ≥ 10	1	0.4	(0.0–1.3)

**Table 6:** Cumulative distribution of the dmf index by individuals and by affected teeth in 12- and 15-year-old students.

	12 Years								15 Years							
	Individuals				DMF Teeth ^b^				Individuals				DMF Teeth			
**DMFT**	***n***	**%**	**Cumulative %**	**95% CI**	***n***	**%**	**Cumulative %**	**95% CI**	***n***	**%**	**Cumulative %**	**95% CI**	***n***	**%**	**Cumulative %**	**95% CI**
≥10	-	-	-		0	-	-		1	0.4	0.4	(0.0–1.3)	12	4.8	4.8	(2.7–7.4)
8	-	-	-		0	-	-		0	-	-		0	-	-	
7	-	-	-		0	-	-		0	-	-		0	-	-	
6	1	0.4	0.4	(0.0–1.3)	6	4.5	4.5	(1.0–8.1)	3	1.3	1.7	(0.0–2.7)	18	7.2	12.0	(4.0–10.4)
5	2	0.9	1.3	(0.0–2.1)	10	7.6	12.1	(3.1–12.1)	8	3.4	5.8	(1.1–5.8)	40	15.9	27.9	(11.4–20.5)
4	9	3.9	5.2	(1.4–2.8)	36	27.3	39.3	(19.7–34.9)	12	5.2	10.9	(2.3–8.0)	48	19.1	47.0	(14.3–24.0)
3	8	3.5	8.7	(1.1–5.8)	24	18.2	57.5	(11.6–24.8)	12	5.2	16.1	(2.3–8.8)	36	14.3	61.4	(10.0–18.7)
2	14	6.1	14.8	(3.0–9.0)	28	21.2	78.7	(14.2–28.2)	27	11.6	27.7	(7.5–15.7)	54	21.5	82.9	(16.4–26.6)
1	29	12.6	27.40	(8.3–16.9)	28	21.2	100.0	(14.2–28.2)	43	18.5	46.1	(13.5–23.4)	43	17.1	100.0	(12.5–21.8)
0	167	72.6	100.0	(66.6–78.49	0	0.0	100.0		127	54.5	100.0	(48.1–60.9)	0	0.0		
Total	230	100			132	10.0			233	100.0			251	100.0		

At age 12, 14.8% of the students (*n* = 230) have 78.7% of the DMF teeth (*n* = 134), or at age 15, 27.7% of the students (*n* = 233) have 82.9% of the DMF teeth (*n* = 251). ^b^ Teeth with a history of caries. (N, sample size; dmf, decayed, missing, and filled primary teeth; CI, confidence interval; DMF Teeth, decayed, missing, and filled permanent teeth).

**Table 7:** Maximum community periodontal index (N, sample size; CPI; community periodontal index; SD: standard deviation; CI, confidence interval; X, excluded sextant).

	12 Years					15 Years				
Maximum CPI	*n*	%	Mean	SD	95% CI	*n*	Mean	SD	%	95% CI
12 years	206					194				
0 (Healthy)	69	30.0	3.63	2.18	(25.3–37.6)	53	2.97	2.26	22.7	(20.7–33.0)
1 (Bleeding)	68	29.6	1.50	1.80	(24.9–37.1)	54	1.97	1.92	23.2	(21.1–33.6)
2 (Calculus)	69	30.0	0.55	1.10	(25.3–37.6)	87	0.66	0.99	52.8	(37.2–51.0)
Not collected	13	-			-	3			-	-
The 6 sextants are X	0	-			-	0			-	-

**Table 8:** Maximum community periodontal index (CPI). Mean number of sextants in each code. (N, sample size; CPI; community periodontal index; SD, standard deviation; CI, confidence interval; X, excluded sextant. Student’s *t*-test, * variable with significant effect (*p* < 0.05).

CPI Code	Sex	12 Years				15 Years			
		N	Mean ± SD	(95%CI)	*p*	N	Mean ± SD	(95%CI)	*p*
0 (Healthy)	Boys	119	3.97 ± 2.02	(3.60–4.34)	0.063	110	2.70 ± 2.29	(2.26–3.13)	0.029 *
	Girls	98	3.53 ± 2.23	(3.08–3.97)		120	3.26 ± 2.21	(2.86–3.66)	
≥1 (Bleeding)	Boys	119	1.21 ± 1.56	(0.93–1.50)	0.004 *	110	2.12 ± 1.92	(1.76–2.49)	0.161
	Girls	98	1.86 ± 2.00	(1.46–2.26)		120	1.87 ± 1.93	(1.52–2.22)	
≥2 (Calculus)	Boys	119	0.67 ± 1.17	(0.46–0.89)	0.053	110	0.71 ± 1.05	(0.51–0.91)	0.261
	Girls	98	0.42 ± 1.00	(0.22–0.63)		120	0.63 ± 0.95	(0.46–0.08	
x (Excluded)	Boys	119	0.00 ± 0.00	-	-	110	0.01 ± 0.19	(0.00–0.05)	-
	Girls	98	0.03 ± 0.22	(0.00–0.07)		120	0.00 ± 0.00	-	
Not collected	Boys	(6)	0.08 ± 0.09	(0.00–0.02)	-	(2)	0.04 ± 0.15	(0.00–0.11)	-
	Girls	(7)	0.03 ± 0.30	(0.00–0.09)		(1)	0.00 ± 0.00	-	
The 6 sextants are X	Boys	0	-	-	-	0	-	-	-
	Girls	0	-	-		0	-	-	

**Table 9:** Prevalence of obesity in students (% according to sex and age). (N, sample size; CI, confidence interval).

		Sex	N	%	95%CI
5–6 years	Severe underweight	Boys (142)	1	0.70	(0.00–2.07)
		Girls (109)	0	0.00	-
	Underweight	Boys (142)	1	0.70	(0.00–2.07)
		Girls (109)	0	0.00	-
	Normal weight	Boys (142)	77	54.23	(46.03–62.41)
		Girls (109)	74	67.89	(59.12–76.65)
	Overweight	Boys (142)	36	25.35	(18.19–32.50)
		Girls (109)	20	18.35	(11.08–25.46)
	Obesity	Boys (142)	27	19.01	(12.55–25.46)
		Girls (109)	15	13.76	(7.29–20.22)
12 years	Severe underweight	Boys (125)	0	0	-
		Girls (102)	1	0.98	(0.00–2.89)
	Underweight	Boys (125)	2	1.60	(0.00–3.79)
		Girls (102)	4	3.92	(0.15–7.68)
	Normal weight	Boys (125)	67	53.60	(44.85–62.34)
		Girls (102)	55	53.92	(44.24–63.59)
	Overweight	Boys (125)	33	26.40	(18.67–34.12)
		Girls (102)	26	25.49	(17.03–33.94)
	Obesity	Boys (125)	23	18.40	(11.60–25.19)
		Girls (102)	16	15.69	(8.62–22.74)
15 years	Severe underweight	Boys (112)	2	1.79	(0.00–4.23)
		Girls (120)	0	0	-
	Underweight	Boys (112)	1	0.89	(0.00–2.63)
		Girls (120)	1	0.83	(0.00–2.45)
	Normal weight	Boys (112)	74	66.07	(57.3–74.8)
		Girls (120)	85	70.83	(62.7–78.9)
	Overweight	Boys (112)	27	24.11	(16.1–32.02)
		Girls (120)	24	20.00	(9.99–23.3)
	Obesity	Boys (112)	8	7.14	(2.37–11.91)
		Girls (120)	10	8.33	(3.38–13.27)

**Table 10:** Anthropometric measurements. Mean, SD, and percentiles. (SD: Standard deviation; CI: confidence interval).

					Percentiles				
		MEAN	SD	95% CI	5	25	50	75	95
Weight (Kg)	5–6 years	25.1	7.63	(24.24–26.14)	18.1	21.5	23.9	27.0	34.3
	12 years	45.63	11.85	(44.10–47.21)	30.8	37.6	43.5	51.6	30.8
	15 years	62.3	12.06	(60.80–64.50)	46.1	54.2	60.7	68.1	84.5
Height (cm)	5–6 years	120.2	6.37	(119.52–121.09)	110.0	116.0	120.0	124.5	130.5
	12 years	150.4	7.93	(149.33–151.40))	137.5	145.0	150.0	156.0	164.3
	15 years	165.9	13.73	(164.20–167.76)	154.2	161.0	166.0	172.3	182.6
BMI (Kg/m^2^)	5–6 years	17.0	2.49	(16.75–17.38)	14.3	15.3	16.6	18.0	21.8
	12 years	19.9	3.96	(19.47–17.38)	54.5	61.5	67.0	74.75	89.0
	15 years	22.3	4.00	(21.85–22.89)	17.41	19.69	21.81	24.18	30.18
Waist circumference (cm)	5–6 years	58.7	8.92	(57.61–59.84)	50.00	53.00	56.50	61.00	77.00
	12 years	68.4	11.28	(66.90–69.90)	65.50	75.25	82.00	89.75	103.0
	15 years	74.46	12.1	(72.89–76.03)	61.00	67.50	72.00	79.25	95.45
Hip circumference (cm)	5–6 years	66.6	6.62	(65.88–67.53)	57.00	62.00	66.00	70.00	80.00
	12 years	82.2	12.25	(80.56–83.75)	13.00	14.00	15.00	16.00	18.00
	15 years	93.01	13.58	(91.25–94.77)	73.78	88.00	93.00	99.60	108.0
Waist-hip ratio	5–6 years	0.88	0.09	(0.86–0.89)	0.77	0.83	0.87	0.91	1.10
	12 years	0.84	0.12	(0.82–0.85)	0.79	0.89	0.83	0.87	1.03
	15 years	0.84	0.54	(0.77–0.91)	0.67	0.74	0.78	0.83	1.15
Waist-to-height ratio	5–6 years	0.49	0.89	(0.47–0.49)	0.42	0.45	0.57	0.51	0.64
	12 years	0.45	0.08	(0.44–0.46)	0.37	0.42	0.45	0.50	0.59
	15 years	0.447	0.07	(0.43–0.45)	0.37	0.40	0.44	0.47	0.57

**Table 11:** Association between overweight/obesity and oral pathology (Adjusted odds ratio analysis). (NA, not applied because periodontal disease is not considered at age 5–6; OR, Odds ratio; CI, confidence interval; Adjusted Odds ratios analysis (95% confidence intervals) *p* < 0.05).

				Caries			Periodontal Disease		
				OR	(95%CI)	*p*	OR	(95%CI)	*p*
			Normal	1.00	(Reference)	-	1	(Reference)	-
			Overweight/obesity	1.179	(0.715–1.943)	0.518	1.085	(0.698–1.686)	0.714
Factors (Potential confounding variables)	Sex	Male	Normal	1.00	(Reference)	-	1	(Reference)	-
			Overweight/obesity	1.198	(0.734–1.955)	0.470	1.360	(0.732–2.525)	0.329
		Female	Normal	1.00	(Reference)	-	1	(Reference)	-
			Overweight/obesity	1.145	(0.700–1.872)	0.590	0.890	(0.470–1.685)	0.721
	Age	5–6 years	Normal	1.00	(Reference)	-	1	(Reference)	-
			Overweight/obesity	1.147	(0.445–2.964)	0.776	NA	NA	NA
		12 years	Normal	1.00	(Reference)	-	1	(Reference)	-
			Overweight/obesity	1.063	(0.588–1.920)	0.841	1.114	(0.614–2.020)	0.723
		15 years	Normal	1.00	(Reference)	-	1	(Reference)	-
			Overweight/obesity	1.756	(0.995–3.099)	0.051	1.256	(0.631–2.500)	0.516
	Area of residency	Urban + Peri-urban	Normal	1.00	(Reference)	-	1	(Reference)	-
			Overweight/obesity	1.390	(0.850–2.271)	0.188	0.774	(0.413–1.453)	0.425
		Rural	Normal	1.00	(Reference)	-	1	(Reference)	-
			Overweight/obesity	0.922	(0.564–1.508)	0.747	1.478	(0.790–2.765)	0.220
	Type of school	Public	Normal	1.00	(Reference)	-	1	(Reference)	-
			Overweight/obesity	0.987	(0.667–1.460)	0.946	0.990	(0.603–1.626)	0.968
		Charter/ private	Normal	1.00	(Reference)	-	1	(Reference)	-
			Overweight/obesity	1.954	(0.917–4.163)	0.079	1.543	(0.577–4.125)	0.385

## Data Availability

The datasets generated and/or analyzed during the current study are not publicly available because we are working on them for other aspects; however, they are available from the corresponding author upon reasonable request.
